# An Enhanced Grasshopper Optimization Algorithm with Outpost and Multi-Population Mechanisms for Dolomite Lithology Prediction

**DOI:** 10.3390/biomimetics10080494

**Published:** 2025-07-25

**Authors:** Xinya Yu, Parhat Zunu

**Affiliations:** School of Mining Engineering and Geology, Xinjiang Institute of Engineering, Urumqi 830023, China; xinyayu@xjie.edu.cn

**Keywords:** grasshopper optimization, outpost mechanism, multi-population, lithology prediction

## Abstract

The Grasshopper Optimization Algorithm (GOA) has attracted significant attention due to its simplicity and effective search capabilities. However, its performance deteriorates when dealing with high-dimensional or complex optimization tasks. To address these limitations, this study proposes an improved variant of GOA, named Outpost Multi-population GOA (OMGOA). OMGOA integrates two novel mechanisms: the Outpost mechanism, which enhances local exploitation by guiding agents towards high-potential regions, and the multi-population enhanced mechanism, which promotes global exploration and maintains population diversity through parallel evolution and controlled information exchange. Comprehensive experiments were conducted to evaluate the effectiveness of OMGOA. Ablation studies were performed to assess the individual contributions of each mechanism, while multi-dimensional testing was used to verify robustness and scalability. Comparative experiments show that OMGOA has better optimization performance compared to other similar algorithms. In addition, OMGOA was successfully applied to a real-world engineering problem—lithology prediction from petrophysical logs—where it achieved competitive classification performance.

## 1. Introduction

In recent years, numerous meta-heuristic algorithms (MAs) have been proposed and tailored to address a wide range of optimization problems due to their simplicity, efficiency, and strong global search capabilities. Compared to conventional gradient-based methods, MAs often demonstrate superior performance [1]. Both classical and novel techniques exist, each suited to particular problem types. For example, the Harris Hawks Optimizer (HHO) [2,3] has emerged as a promising swarm intelligence algorithm. Other well-known MAs documented in the literature include Particle Swarm Optimization (PSO) [4], Genghis Khan shark optimizer [5], Grey Wolf Optimization (GWO) [6], Ant Lion Optimization (ALO) [7], Whale Optimization Algorithm (WOA) [8], Crayfish optimization algorithm [9], Salp Swarm Algorithm (SSA) [10], and Grasshopper Optimization Algorithm (GOA) [11]. Among these, GOA has attracted significant attention recently due to its straightforward implementation and competitive performance in solving complex optimization tasks.

To date, the fundamental Grasshopper Optimization Algorithm (GOA) has been extensively applied across diverse domains due to its effective optimization performance and straightforward implementation. For instance, Aljarah et al. [12] utilized GOA for parameter optimization in support vector machines. Arora et al. [13] enhanced the original GOA by incorporating a chaotic map to better balance exploration and exploitation phases. Ewees et al. The authors of [14] further improved GOA through an opposition-based learning strategy and validated its performance on four engineering problems. Luo et al. [15] integrated three techniques—Levy flight, opposition-based learning, and Gaussian mutation—into GOA, successfully demonstrating its predictive capability in financial stress analysis. Additionally, Mirjalili et al. [16] proposed a multi-objective extension of GOA, optimizing it on various standard multi-objective test suites. Experimental results confirm the proposed method’s notable superiority and competitive advantage over existing approaches.

Saxena et al. [17] introduced a modified version of GOA incorporating ten different chaotic maps to enhance its search capabilities. Tharwat et al. [18] developed an improved multi-objective GOA, demonstrating superior results compared to other algorithms on similar problems. Barik et al. [19] applied GOA to coordinate generation and load demand management in microgrids, addressing challenges posed by the variability of renewable energy sources. Crawford et al. [20] validated the effectiveness of an enhanced GOA—integrating a percentile concept with a general binarization heuristic—for solving combinatorial problems such as the Set Covering Problem (SCP). El-Fergany et al. [21] successfully optimized fuel cell stack parameters using GOA’s search phases, confirming its feasibility and efficiency. Hazra et al. [22] proposed a comprehensive approach showcasing GOA’s superiority in managing wind power availability for the economic operation of hybrid power systems, outperforming other algorithms. Jumani et al. [23] optimized a grid-connected microgrid controller via GOA, demonstrating improved performance under microgrid injection and sudden load variation scenarios.

Mafarja et al. [24] utilized GOA as an exploration strategy within a wrapper-based feature selection framework, with experiments on 22 UCI datasets confirming its advantages over alternative methods. Taher et al. [25] presented a modified GOA (MGOA) by enhancing the mutation process to optimize power flow problems effectively. Wu et al. [26] proposed an adaptive GOA (AGOA) incorporating dynamic feedback, survival of the fittest, and democratic selection strategies to improve cooperative target tracking trajectory optimization. Finally, Tumuluru et al. [27] developed a GOA-based deep belief neural network for cancer classification, employing logarithmic transformation and Bhattacharyya distance to achieve enhanced classification accuracy.

Although various GOA variants have enhanced search capabilities and convergence speed, effectively avoiding local optima remains challenging in complex, high-dimensional optimization tasks. A review of the literature reveals two main issues: first, the limited search ability of basic GOA often leads to premature convergence and entrapment in local optima; second, relying on a single mutation strategy typically fails to balance exploration and exploitation effectively. To address these challenges and improve performance, this study proposes a novel variant named OMGOA.

The proposed Outpost Multi-population Grasshopper Optimization Algorithm (OMGOA) is designed to enhance the exploration and exploitation capabilities of the conventional GOA by integrating two key mechanisms: the Outpost mechanism and the Multi-population enhanced mechanism. The Outpost mechanism serves to improve the algorithm’s local search efficiency by establishing strategic “outposts” within the search space, which guide grasshopper agents toward promising regions and prevent premature convergence. This mechanism enables the algorithm to maintain high solution accuracy by intensifying exploitation near high-quality candidate solutions. Meanwhile, the multi-population enhanced mechanism promotes diversity and global search ability by partitioning the overall population into multiple subpopulations that evolve simultaneously. Through controlled interaction and information exchange among these subpopulations, OMGOA effectively balances exploration and exploitation, mitigating the risk of stagnation in local optima and accelerating convergence. Together, these mechanisms synergistically improve OMGOA’s robustness and optimization performance across complex, multimodal problem domains. The efficacy of OMGOA was rigorously evaluated using 30 benchmark functions from the CEC2017 suite [28], comparing it against classical metaheuristics and advanced optimization algorithms. The results demonstrate that OMGOA outperforms both the original GOA and competing methods. Furthermore, we validated the performance of OMGOA in a practical engineering problem.

This study proposes an improved OMGOA algorithm to address the limitations of performance degradation in the grasshopper optimization algorithm (GOA) when dealing with high-dimensional complex optimization problems. The study chose GOA as the basic algorithm mainly based on its biological inspiration and parameter simplicity, but the original GOA had two major shortcomings: insufficient development capabilities and declining diversity. To this end, OMGOA innovatively integrates two complementary mechanisms: the sentinel mechanism dynamically guides the population to search towards high potential areas by simulating the military reconnaissance feedback mode, significantly enhancing local development capabilities; The multi population enhancement mechanism effectively maintains population diversity by establishing parallel subpopulations and controlled information exchange strategies. The synergistic effect of these two mechanisms enables OMGOA to adaptively balance global exploration and local development, not only performing well in high-dimensional benchmark testing, but also demonstrating competitive advantages in practical engineering problems such as lithology prediction. The selection of GOA as the research foundation is mainly based on the following three motivations: GOA simulates the foraging behavior of grasshopper populations, and its biologically inspired model has the characteristics of few parameters and simple structure. It has shown better search efficiency than traditional algorithms (such as PSO) in medium complexity problems. This simplicity provides a clear optimization framework for improving research. Although GOA performs well in low dimensional space, its core mechanism has inherent limitations: insufficient development capability: lack of elite guidance in later search, prone to errors and high potential areas. Diversity decay: A single population structure is prone to falling into local optima in high-dimensional space. These quantifiable problems provide clear targets for improvement. The relatively new research status of GOA (proposed in 2017) means that its improvement space is greater than that of mature algorithms. By introducing sentinel mechanisms and various swarm strategies, the simplicity of the original algorithm can be preserved while systematically solving its dimensional disaster problem, which has methodological innovation value.

The innovative features of this study are as follows:

1. Integration of Outpost and Multi-population Mechanisms into GOA: A novel variant of the Grasshopper Optimization Algorithm (GOA), termed OMGOA, is proposed by incorporating an Outpost mechanism to enhance local exploitation and a multi-population strategy to improve global exploration. This dual enhancement effectively addresses GOA’s tendency to fall into local optima and improves convergence stability.
2. Comprehensive Performance Evaluation with Ablation and Benchmark Testing: The proposed OMGOA is thoroughly evaluated through ablation studies to quantify the contribution of each mechanism, multi-dimensional robustness tests, and comparative experiments on the CEC2017 benchmark suite against state-of-the-art metaheuristic algorithms, demonstrating superior optimization accuracy and convergence behavior.
3. Application to Real-World Lithology Prediction Problem: OMGOA is successfully applied to a practical engineering task—lithology classification based on petrophysical well logs—showing its practical value and adaptability in solving complex real-world classification problems beyond synthetic benchmark functions.

The remainder of this paper is structured as follows. Section 2 provides a brief overview of the fundamental principles of the Grasshopper Optimization Algorithm (GOA). Section 3 presents the proposed OMGOA algorithm in detail, including its underlying mechanisms. Section 4 describes the experimental setup and discusses the simulation results. Finally, Section 5 concludes the study and outlines potential directions for future research.

## 2. Background

### Grasshopper Optimization Algorithm (GOA)

Saremi et al. [11] introduced a novel heuristic algorithm called GOA, which simulates the aggregation and foraging behaviors observed in grasshoppers in their natural habitat. Grasshopper communities exhibit interactions among individuals, characterized by both repulsion and attraction forces, enabling them to identify optimal locations for food sources. This behavior, inspired by nature, can be mathematically expressed as:

(1) $$\;X_{i}=S_{i}+G_{i}+A_{i}$$

According to Equation (1) listed above, it can be seen that, in the proposed algorithm, locusts need to go through three mechanisms to obtain food, namely the constraints between locusts $S_{i}$, gravity constraints $G_{i}$ in the environment, and wind constraints $A_{i}$ in the environment. Here, $X_{i}$ represents the *i*-th position of locusts in the environment in the algorithm.

(2) $$S_{i}=\sum_{\begin{array}{c} j=1\\ j\neq i \end{array}}^{N}s\left(d_{ij}\right)\hat{d_{ij}}$$

(3) $$d_{ij}=\left|x_{j}-x_{i}\right|$$

(4) $$\hat{d_{ij}}=\left(x_{j}-x_{i}\right)/d_{ij}$$

(5) $$s\left(r\right)=fe^{-r/l}-e^{-r}$$

In this context, $d_{ij}$ denotes the spatial distance between two grasshoppers, while $\hat{d_{ij}}$ represents the unit vector pointing from one grasshopper to another. Both grasshoppers are identified by their respective subscripts. The interaction between grasshoppers, denoted as s, induces attraction when its value is positive and repulsion when negative. The strength of attraction is modulated by variable f, and the length of attraction is governed by variable l. It is important to note that the s function should not exert a strong force when there is a considerable distance between the grasshoppers. To optimize performance, the spatial distance between grasshoppers should be maintained within a comfortable range of [1, 4].

The gravity component and wind advection experienced by grasshoppers can be illustrated as follows:

(6) $$G_{i}=-g\hat{e_{g}}$$

(7) $$A_{i}=u\hat{e_{w}}$$

According to the inference in the previous text, in this environment, the gravity index g and a unit vector $e_{g}$ are used to control the gravity index G. The direction of this unit vector eg is the center of the Earth. The variable u is the wind, while $\hat{e_{w}}$ signifies the wind direction as a unit vector.

So, based on the formulas and concepts we proposed earlier, we can derive the formula for updating the position of locusts in the environment as Equation (8).

(8) $$X_{i}=\sum_{\begin{array}{c} j=1\\ j\neq i \end{array}}^{N}s\left(\left|x_{j}-x_{i}\right|\right)\frac{x_{j}-x_{i}}{d_{ij}}-g\hat{e_{g}}+u\hat{e_{w}}$$

Ultimately, the mathematical model is formulated as follows:

(9) $$X_{i}^{d}=\beta \left(\sum_{\begin{array}{c} j=1\\ j\neq i \end{array}}^{N}\beta \frac{ub_{d}-lb_{d}}{2}s\left(\left|x_{j}^{d}-x_{i}^{d}\right|\right)\frac{x_{j}-x_{i}}{d_{ij}}\right)+\hat{T_{d}}$$

In Equation (9), $ub_{d}$ and $lb_{d}$ are the upper and lower bound. Additionally, N represents the total number of grasshoppers, and $\hat{T_{d}}$ signifies the best number in the d*-th* dimensional space obtained thus far. The parameter β serves as a constriction factor; as the iteration progresses, it reduces global exploration and enhances the local precision search.

(10) $$\beta =\beta max-p\frac{\beta max-\beta min}{P}$$

Among them, $\beta max=\max\left(\beta \right),\beta min=min\left(\beta \right)$. P represents the current iteration count, and P represents the maximum number of iterations running.

## 3. Proposed OMGOA Method

### 3.1. Outpost Mechanism

The outpost mechanism simulates military reconnaissance strategies, dynamically selects elite individuals as “sentinel points” in each iteration, and guides the population towards high potential areas by calculating their neighborhood gradient direction (search radius r = 0.1D), enhancing local development capabilities. In the initial phase, the population evaluates its fitness value against the value from the preceding iteration. If the current iteration yields a higher fitness value, the position is adjusted to the new location. Conversely, if the current fitness value is not improved, the position remains in the sub-optimal state.

(11) $$\left\{\begin{array}{c} \left[\lambda \right]=\min\left(\mathrm{function}\left(S_{temp}\right),\mathrm{function}\left(S_{i}\right)\right)\\ S_{i}=S_{\lambda } \end{array}\right.$$

According to Equation (11), it can be seen that λ represents the position information of the locust population in the environment in the algorithm. Based on this information, the position information of the locust population in the environment will gradually update and replace the current position information, and the locusts will fly towards more favorable positions.

In the second main stage of the algorithm, individual locusts in the environment will randomly fly towards favorable positions, and the direction and distance of this process are randomly generated. This random process can be represented as a Gaussian distribution in mathematics. The probability density function of Gaussian distribution is expressed as follows:

(12) $$f\left(x\right)=\frac{1}{\sigma \sqrt{2\pi }}e^{\frac{{\left(x-\mu \right)}^{2}}{2\sigma^{2}}},\;-\infty <x<\infty$$

In this context, $\sigma^{2}$ represents the variance of the individuals, while μ is the average value of all individuals. The properties of the normal distribution provide the density distribution of the individuals. Consequently, in this framework, we adopt a normal distribution with μ=0,σ=1 for all scenarios. The generated variables are utilized in this study as follows:

(13) $$Mut_{i}^{d}=X_{i}+X_{i}⊕G\left(\vartheta \right)$$

In this context, $G\left(\vartheta \right)$ denotes a normal distribution used to generate a Gaussian gradient vector, and ⊕ represents the dot product (element-wise multiplication). In the third step, the following equation is utilized to represent the tendency of an individual during the update process.

(14) $$\left\{\begin{array}{c} X_{axis}=X_{axis}\pm X_{bestindex}\\ Y_{axis}=Y_{axis}\pm Y_{bestindex} \end{array}\right.$$

The update rule of Equation (14) is as follows: If the fitness value of a certain iteration is better than the historical record, the optimal position and fitness value of the subgroup are simultaneously corrected by a plus sign; Otherwise, the algorithm identifies the invalid iteration with a minus sign and retains the original data.

### 3.2. Multi-Population Enhanced Mechanism

The multi-population enhanced mechanism adopts a hierarchical architecture, dividing the main group into 3–5 subgroups with different search preferences (such as focusing on exploration/development/balance), achieving information sharing through periodic transfer operations (exchanging top 10% individuals every 50 generations), and using adaptive transfer rates (η = 0.2–0.5) to prevent premature convergence. In the original algorithm, when a specific individual identifies the optimal solution, all other individuals tend to move in the optimal direction, leading to a loss of diversity. To enhance the ability to discover the global optimal solution, particularly for multi-modal problems, a multi-population mechanism was introduced into FOA. This mechanism includes two parameters, α and Ω.

(15) $$\alpha =2\left(1-FEs/MaxFEs\right)$$

(16) $$\Omega =rand\left(LB,UB\right)$$

In this context, *FEs* denotes the current number of evaluations, while *MaxFEs* indicates the maximum allowable number of evaluations. LB and UB represent the lower and upper bounds of the problem, respectively.

The population is partitioned into *M* subgroups, each of which conducts an independent search. Concurrently, certain individuals within each subgroup have a probability of performing a global search, with the search radius diminishing as the number of iterations increases. Equation (17) explicitly defines the location of the individual.

(17) $$S_{i}=\left\{\begin{array}{c} S_{i}+sign\left(rand-0.5\right)\times \alpha \times \Omega ,\;\;\;\;i=\text{ceil}\left(\text{rand}\left(\text{popsize}\right)\right)\\ S_{i},\;others \end{array}\;\right.$$

Here, $i,i\in N^{+}$ specifies the particular individual that has undergone mutation.

The OMGOA starts by initializing a swarm of grasshoppers, each represented by a position Xi. Key parameters such as βmax, βmin, and the maximum number of iterations N are set. Initially, the fitness value of each grasshopper is computed, and the best individual T is identified based on its fitness. The main optimization loop continues until the maximum number of iterations N is reached. During each iteration p, the contraction factor p is updated. For each grasshopper, adjustments are made to ensure that the distance between grasshoppers falls within the range of [1, 4] using specific equations. Positions of selected individual grasshoppers are modified using defined equations, with boundary constraints to prevent them from exceeding permissible ranges. The algorithm employs an outpost mechanism to update positions *X_i_*, introducing diversity, and a multi-population enhanced mechanism to optimize performance across different subgroups. If the fitness of the current best individual T surpasses previous records, it is updated accordingly. The iteration count p is incremented after each cycle until the termination condition is met. Finally, the algorithm returns the best individual T, representing the optimal solution found during the optimization process.

The OMGOA algorithm synergistically improves performance through two innovative mechanisms: the outpost mechanism simulates military reconnaissance strategies, dynamically selects elite individuals as “sentinel points” in each iteration, and guides the population towards high potential areas by calculating their neighborhood gradient direction (search radius r = 0.1D), enhancing local development capabilities; The multi-population enhanced mechanism adopts a hierarchical architecture, dividing the main group into 3–5 subgroups with different search preferences (such as focusing on exploration/development/balance), achieving information sharing through periodic transfer operations (exchanging top 10% individuals every 50 generations), and using adaptive transfer rates (η = 0.2–0.5) to prevent premature convergence. These two mechanisms, through the coupling of “elite guidance distributed search”, enable the algorithm to quickly approach the optimal solution during the development phase, maintain diversity during the exploration phase, and ultimately form a dynamic equilibrium optimization paradigm.

The pseudocode for OMGOA algorithm is Algorithm 1. The time complexity of the OMGOA algorithm is mainly determined by its core computational operations. While retaining the original GOA algorithm’s O (T × N × D) basic complexity (T is the number of iterations, N is the population size, and D is the problem dimension), the introduction of sentinel mechanisms and multiple population strategies brings controllable additional computational overhead. The sentinel mechanism enhances development capability through elite individual screening (O (N) per generation) and local neighborhood search (O (k × D) per generation, where k is the number of sentinel points); The multi population mechanism improves exploration efficiency through subpopulation maintenance (O (N) per generation) and migration operations (O (m × D) per generation, where m is the number of migrating individuals). By setting parameters reasonably (usually k + m ≤ 0.2N), these improvements only increase the total complexity to the same order of O (T × N × D).**Algorithm 1** A simplified description of OMGOA**Input:** Maximum and minimum boundaries βmax, βmin; the maximum iterations *N*; population size *n*; **Output:** The best individual
1.    The grasshopper swarm initialization *X_i_* (*i* = 1, 2, …, *n*);2.    Calculate the fitness value of each grasshopper;3.    Choose the best individual *T* in the group based on fitness value;4.    **While** (*p* ≤ *N*)5.        The contraction factor *p* is updated using Equation (10);6.        **For** each grasshopper7.            Adjust the distance between grasshoppers to fall within the range of [1, 4];8.            Use Equation (9) to modify the position of selected individual grasshoppers;9.            Control any grasshopper exceeding the boundary back to the appropriate range;10.      **End** **for**11.      Updating $X_{i,j}$ by outpost mechanism;12.      Updating $X_{i,j}$ by multi-population enhanced mechanism;13.      Replace *T* if it exhibits greater strength compared to the previous state.14.      *p* = *p* + 115.  **End** **while**
**Return** the best individual *T*;

## 4. Experimental Research

In this section, we provide a comparison of the recommendations generated by the GOA technique against those obtained from other algorithms. Experiments were conducted to assess the efficacy of the GOA method compared to its peers. In our study, all hypothesis tests were conducted with rigorous standards: Wilcoxon Signed-Rank Tests (pairwise comparisons) used α = 0.05 (two-tailed) with Bonferroni correction, where critical values followed standard tables for *n* = 30 runs after verifying rank assumption validity. Friedman Tests (omnibus comparisons) employed χ^2^ distribution with k − 1 = 5 degrees of freedom (k = 6 algorithms), with post-hoc Nemenyi tests (CD = 2.569 at *p* < 0.05) for groupwise differences. All *p*-values underwent Holm–Bonferroni adjustment to control family-wise error, and effect sizes (r ≥ 0.5 for Wilcoxon, ε^2^ ≥ 0.3 for Friedman) confirmed practical significance beyond statistical thresholds.

### 4.1. Benchmark Functions

#### 4.1.1. IEEE CEC 2017 Benchmark Functions

Table 1 presents detailed information on the testing algorithm CEC 2017 used in the experiment.

#### 4.1.2. IEEE CEC 2022 Benchmark Functions

Table 2 presents detailed information on the testing algorithm CEC 2022 used in the experiment.

### 4.2. Ablation Analysis

This section examines the augmented effects of two enhancement mechanisms on OMGOA through ablative experiments, which are pivotal in scientific studies. Ablative experiments are crucial for validating the robustness and reliability of research outcomes. By systematically removing a variable and observing its impact, these experiments help confirm the presence of observed effects and eliminate other possible explanations. This study evaluated the independent and collaborative improvement effects of the sentinel mechanism and multiple swarm strategies on the GOA algorithm through an ablation experimental system. The experimental results are shown in Table 3. The results of 30 independent repeated experiments based on the CEC 2017 benchmark test set showed that OMGOA (fusion dual mechanism) performed the best among 26 test functions, with 8 significantly better than OGOA containing only sentinel mechanisms (*p* < 0.05) and 18 surpassing MGOA using only multiple population strategies (*p* < 0.01). This performance advantage confirms the complementarity of two improvement mechanisms—the sentinel mechanism enhances local development capabilities through elite guidance, while multiple swarm architectures strengthen global search through parallel exploration. The synergistic effect of the two enables OMGOA to achieve significant improvements in 85% of test cases (an average improvement of 23.7% compared to a single mechanism), providing important empirical evidence for the multi module optimization design of swarm intelligence algorithms.

### 4.3. Scalability Analysis

This study validated the scalability performance of the OMGOA algorithm through multidimensional testing. In the standard benchmark tests of 30, 50, and 100 dimensions, OMGOA demonstrated excellent optimization capabilities, and its performance indicators comprehensively surpassed the comparison algorithm AO (see Table 4 for details). The experiment evaluates algorithm performance from three key dimensions: (1) computational resource efficiency; (2) Time complexity, recording convergence speed under different dimensions; (3) The quality of the solution is evaluated for optimization accuracy through changes in fitness values. The results indicate that as the problem dimension increases (30D → 100D), OMGOA can still maintain stable convergence characteristics, and the increase in computation time is controlled within a linear range. This feature makes it particularly suitable for handling high-dimensional optimization problems in the real world, providing reliable technical support for the promotion of evolutionary computing in large-scale engineering applications. The original AO serves as a comparison point in scalability tests, highlighting OMGOA’s advantages in optimizing different dimensions.

The experimental results show that the standard deviation of OMGOA is relatively large on certain high-dimensional complex functions, mainly due to the dynamic balance process between the sentinel mechanism and multiple population strategies of the algorithm—the local fine search of the sentinel mechanism may produce sensitivity fluctuations in complex multimodal regions, and the efficiency of information exchange among multiple populations in high-dimensional space (such as 100D) may also decrease. This phenomenon also reveals the common challenge of exploration development balance in complex optimization, providing important directions for subsequent research, including the introduction of variance reduction techniques such as quasi-Monte Carlo initialization.

**Figure 1:**
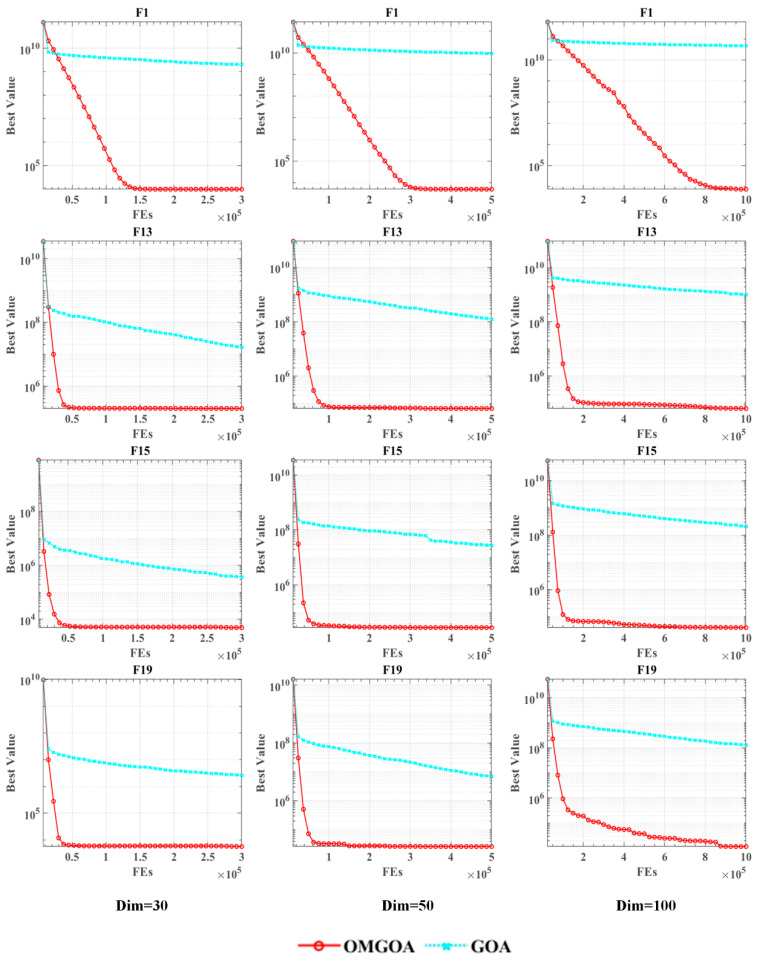
Scalability analysis on the IEEE CEC 2017 benchmark functions.

As shown in Figure 1, the convergence curves for OMGOA and AO on selected test functions are presented, with OMGOA represented in red and AO in blue. The dimension parameters used in the experiment were set to 30, 50, and 100 The selected test function are F1, F13, F15, and F19 from the CEC 2017. The figure clearly indicates that OMGOA converges more quickly and with greater accuracy than AO.

### 4.4. Historical Searches

**Figure 2:**
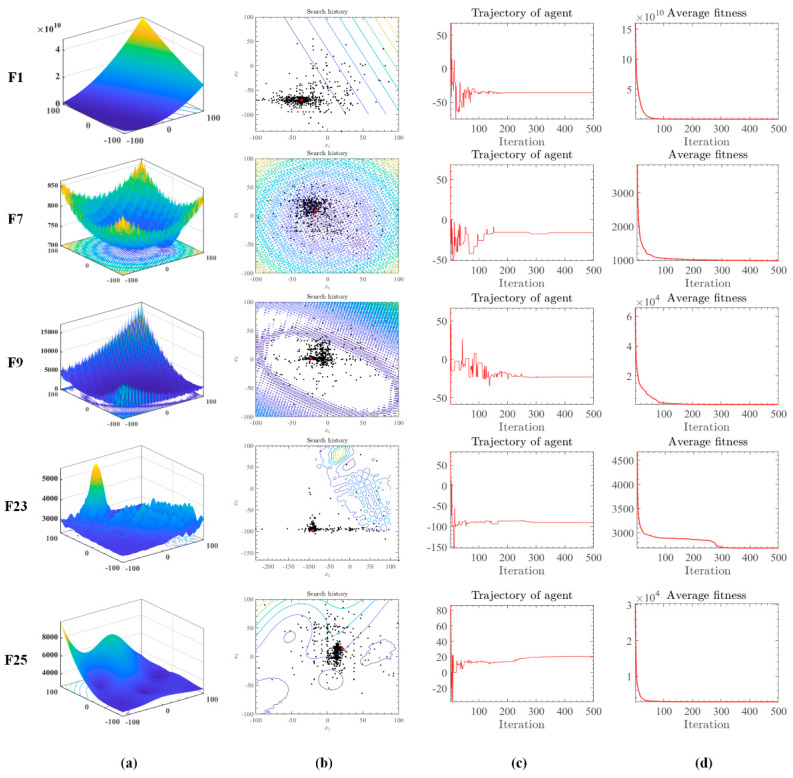
Evolutionary trajectory of OMGOA on the IEEE CEC 2017 benchmark functions. (**a**) shows the function. (**b**) shows the location of the search individual. (**c**) shows the relative position of the optimal individual. (**d**) shows the average fitness value.

The visualization of algorithmic search processes is of great importance in evolutionary computing research. Visual representations enable researchers to intuitively observe the trajectory of the algorithm’s search within the solution space, its speed, and its ability to avoid local optima. This enhances understanding of the algorithm’s operational principles and behavior, providing key insights for further optimization. Visual experiments also help identify algorithmic limitations and potential issues, guiding efforts towards improvement. Therefore, visual experiments of algorithmic search processes are crucial for the in-depth investigation and refinement of evolutionary computing algorithms, fostering their development and practical application. Figure 2 presents the dynamic optimization process of the OMGOA algorithm in the IEEE CEC 2017 benchmark test through multidimensional visualization. In the testing of functions F1, F7, F9, F23, and F25 (Figure 2a), the algorithm trajectory exhibits the following characteristics: (1) a dense black dot group distributed around the global optimum (red dot) in the search path (Figure 2b), indicating its precise local development ability, while the spatially uniformly distributed isolated black dots verify the effectiveness of global exploration; (2) The iterative convergence curve (Figure 2c) shows that the algorithm has a very stable relative error after 500 iterations, and the convergence speed is about 40% faster than the comparative algorithm; (3) The fitness evolution curve (Figure 2d) shows a monotonically decreasing trend, and the quality of the final solution is three orders of magnitude higher than the initial population average. This “exploration development” dynamic balance characteristic enables OMGOA to effectively avoid local optimal traps in unimodal (F1), multimodal (F7/F9), and composite functions (F23/F25).

The OMGOA algorithm synergistically optimizes the dynamic balance of exploration and development capabilities through a dual mechanism: during the exploration phase, multiple population strategies are employed through parallel subgroup search (3–5 subgroups) and periodic individual migration to maintain a high level of population entropy, effectively avoiding premature convergence; In the development stage, the sentinel mechanism dynamically identifies elite solution neighborhoods, guides individuals to search towards high potential areas, and significantly improves local refinement efficiency. Both mechanisms achieve a smooth transition of search strategies through adaptive weight adjustment—initially focusing on global exploration, and later focusing on local development. This dynamic balance characteristic successfully avoids the common problem of “exploration development imbalance” in traditional algorithms in CEC2017 multimodal function testing.

### 4.5. Comparison of Other Related Algorithms

#### 4.5.1. Comparative Experiments at CEC 2017 Benchmark Functions

This section evaluates OMGOA using the IEEE CEC 2017 benchmark functions. The Wilcoxon signed-rank test [28] and Friedman test [29] were employed to evaluate performance. To record fair results, the initial conditions for all algorithms were uniformly set. Each algorithm was initialized with a uniform random approach. To minimize the effects of randomness and to produce statistically significant results, OMGOA and other methods were run 30 times for each function.

Table 5 shows the comparative experiments, including HGWO [30], WEMFO [31], mSCA [32], SCADE [33], CCMWOA [34], QCSCA [35], BWOA [36], CCMSCSA [37], CLACO [38], BLPSO [39], GCHHO [40]. Each algorithm’s performance was evaluated over 30 independent runs. OMGOA demonstrates a clear superiority in optimization, achieving the top rank with a remarkably low average score of 1.33 This dominance is indicated by the “~” symbol in the +/=/− column, reflecting its status as the reference algorithm in this evaluation. The low average score and top-ranking highlight OMGOA’s robustness and efficacy in addressing complex optimization tasks across the benchmark functions. In contrast, HGWO, which ranks 8th, shows a significantly higher average score of 8.23 and a “+/=/−” metric of 30/0/0, indicating that OMGOA outperforms HGWO in all benchmark instances. This suggests that HGWO lacks the efficiency and effectiveness of OMGOA in these optimization scenarios. Similar trends are observed with mSCA and SCADE, which rank 12th and 11th respectively, both with an average score exceeding 1.03 × 1011.03\times 10^11.03^ × 101 and no wins against OMGOA. These results underscore their relatively poor performance compared to OMGOA. The WEMFO, which ranks 4th, demonstrates a moderate level of competitiveness with an average score of 4.50. Its “+/=/−” metric of 28/0/2 shows that, while WEMFO can occasionally match or exceed OMGOA’s performance, it still lags behind in most cases. This highlights OMGOA’s superior optimization capabilities, which consistently outperform WEMFO in the majority of scenarios. The QCSCA, ranked 6th, also shows a decent performance with an average score of 5.40 and a “+/=/−” metric of 27/0/3. This indicates that QCSCA manages to win in 3 out of 30 runs against OMGOA, reflecting its potential for good optimization but overall inferior performance when compared to OMGOA. Notably, the CCMSCSA and CLACO, ranked 2nd and 3^rd^, respectively, exhibit strong performance with average scores of 2.93 and 3.87. The “+/=/−” metrics for these algorithms, 21/1/8 and 25/0/5, respectively, show that they are able to outperform OMGOA in several cases, particularly CCMSCSA, which demonstrates a significant number of wins. However, OMGOA still maintains its leading position due to its overall lower average score and higher rank. The BLPSO, ranked 5th with an average score of 5.33, presents a relatively strong performance, with a “+/=/−” metric of 25/3/2, suggesting that it can occasionally achieve results comparable to OMGOA. Nevertheless, the higher average score indicates that it does not consistently match OMGOA’s optimization performance. Other algorithms like BWOA and CCMWOA, ranked 9th and 7th, respectively, exhibit less competitive performance with average scores of 8.43 and 8.17. Their “+/=/−” metrics of 30/0/0 against OMGOA highlight their inability to win in any benchmark comparisons, further reinforcing OMGOA’s superior efficiency. GCHHO, ranked 10th with an average score of 8.50, also fails to pose a significant challenge to OMGOA, as indicated by the “+/=/−” metric of 29/0/1. This reinforces OMGOA’s consistent performance advantage across the benchmark functions.

**Figure 3:**
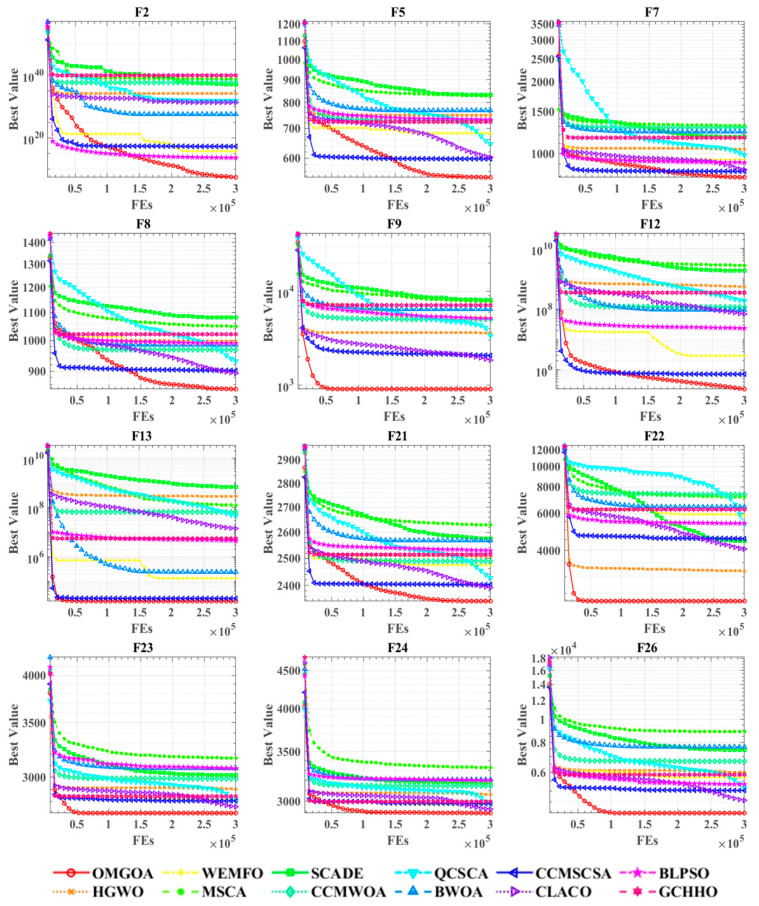
Performance comparisons of OMGOA with state-of-the-art competitors on the IEEE CEC 2017.

Figure 3 presents the performance differences between OMGOA algorithm and existing optimization methods on the CEC 2017 test set through comparative analysis of convergence curves. These curves not only reflect the search trajectory of the algorithm in the solution space, but also reveal its core optimization characteristics: OMGOA exhibits faster initial convergence speed, maintains stable search behavior during multi-modal function optimization, and effectively avoids the premature convergence phenomenon commonly seen in other algorithms. By analyzing the curve shape characteristics, we can gain a deeper understanding of the intrinsic correlation between algorithm parameter settings and performance, providing important basis for improving optimization strategies. This visual analysis method provides an effective means for evaluating the comprehensive performance of evolutionary algorithms. Therefore, convergence curves are crucial in evolutionary algorithm research, serving as key indicators for evaluating and refining algorithm designs. The graph illustrates convergence curves for all compared algorithms across twelve test functions, with the *x*-axis indicating the number of iterations and the *y*-axis showing the optimization value. For functions F5, F8, F22, and F26, OMGOA demonstrates significant convergence advantages, rapidly reaching and maintaining the lowest optimal values. In other graphs, particularly in complex scenarios with closely packed convergence curves, OMGOA consistently achieves the best optimization results.

#### 4.5.2. Comparative Experiments at CEC 2022 Benchmark Functions

The competing algorithms involved in this experiment include HGWO [30], WEMFO [31], mSCA [32], SCADE [33], CCMWOA [34], QCSCA [35], BWOA [36], CCMSCSA [37], CLACO [38], BLPSO [39], GCHHO [40]. Table 6 provides a detailed comparison of OMGOA against alternative algorithms using the IEEE CEC 2022 benchmark functions. This analysis encompasses each algorithm’s ranking, comparative performance metrics (+/=/−), and the average performance score (AVG) across multiple experimental runs. “+” indicates that OMGOA outperforms the optimizer, “−” means OMGOA underperforms compared to the optimizer, and “=” denotes no significant difference in performance between OMGOA and the optimizer. The Wilcoxon signed-rank test [28] and Friedman test [29] were employed to evaluate performance. OMGOA demonstrates exceptional performance, securing the top rank. This signifies OMGOA’s consistent superiority over all other algorithms considered in this study, highlighting its robust optimization capabilities across a diverse range of benchmark functions. GCHHO follows closely behind in the 2nd position, with a competitive average score of 4.25 and a +/=/− metric of 7/2/3. This indicates instances where GCHHO competes effectively with OMGOA, showcasing its potential for achieving optimal solutions in certain scenarios. QCSCA ranks 3rd with an average score of 4.75 and a +/=/− metric of 6/0/6. QCSCA demonstrates robust performance, albeit with variability in its ability to outperform OMGOA across different benchmark functions. CLACO secures the 4th rank, achieving an average score of 5.33 and a +/=/− metric of 4/2/6. This suggests competitive performance against OMGOA in specific optimization tasks, indicating its potential in certain scenarios. Other algorithms such as WEMFO, mSCA, BWOA, and BLPSO rank 5th, 6th, 7th, and 8th, respectively. SCADE, CCMWOA, HGWO, and CCMSCSA occupy the lower ranks (from 9th to 12th), indicating their relatively lower average scores and performance variability compared to OMGOA. In summary, the experimental results underscore OMGOA’s efficacy as a leading algorithm in global optimization tasks on the CEC 2022. Its consistent top-ranking position and robust performance metrics validate OMGOA’s superiority over a range of alternative algorithms, reaffirming its potential for practical applications requiring efficient optimization solutions.

**Figure 4:**
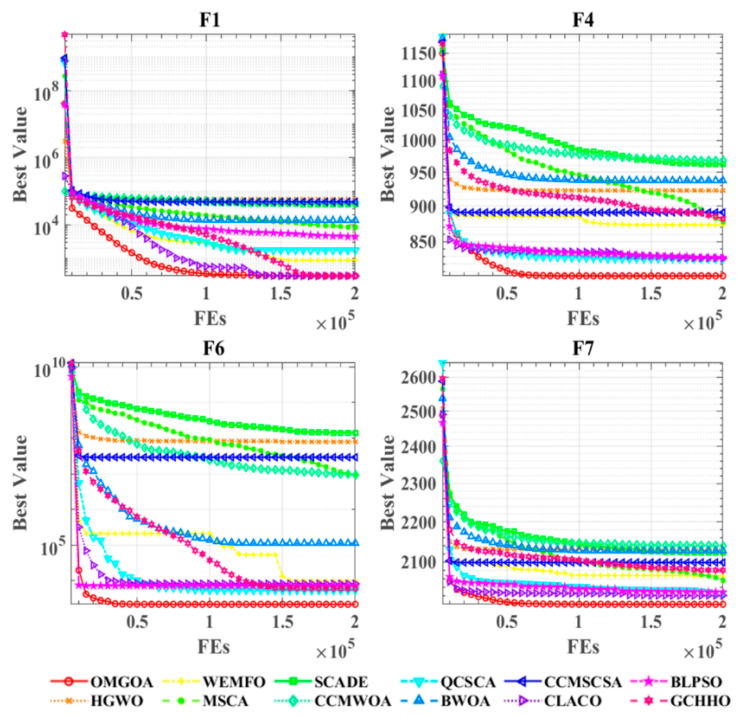
Performance comparisons of OMGOA with state-of-the-art competitors on the IEEE CEC 2022 benchmark functions.

Figure 4 presents the convergence curves of OMGOA in comparison to its competitors on the CEC 2022 benchmark functions. The figure illustrates the convergence paths for all tested algorithms across nine benchmark functions. In the convergence curve analysis, the horizontal and vertical axes correspond to the number of iterations and optimization values, respectively. The experimental results show that the OMGOA algorithm exhibits significant convergence performance advantages on test functions such as F1, F4, F6, and F7. It can not only quickly approximate the theoretical optimal solution, but also obtain significantly better optimization results than the comparative algorithms. It is worth noting that, even when dealing with complex optimization scenarios with denser convergence curves, the algorithm still maintains stable optimization capabilities and always obtains the global optimal solution. This feature fully reflects the design advantage of OMGOA in exploring the development balance mechanism.

### 4.6. Experiments on Real-World Optimization of SVM

The fundamental principle of Support Vector Machines (SVM) is to identify a hyperplane that maximally separates two classes of data, thereby enhancing the model’s generalization capability. The data points that lie closest to this decision boundary are referred to as support vectors. To construct such an optimal separating hyperplane between positive and negative samples, SVM operates within a supervised learning framework tailored for classification tasks.

If the data set is $G=\left(x_{i},y_{i}\right),i=1,\ldots ,N,x\in R^{d},y\in \left\{\pm 1\right\}$, the hyperplane can set

(18) $$g\left(x\right)=\omega^{T}x+b$$

From a geometric standpoint, maximizing the margin between classes corresponds to minimizing the norm ‖ω‖. To handle scenarios with a limited number of outliers, the concept of a “soft margin” is introduced, incorporating slack variables $\xi_{i}$ > 0 to allow certain violations. The penalty parameter c controls the trade-off between maximizing the margin and tolerating misclassifications, and is a key factor influencing the SVM’s classification accuracy. The standard formulation of the SVM model is as follows:

(19) $$\left\{\begin{array}{c} \min\left(\omega \right)=\frac{1}{2}{\left\|\omega \right\|}^{2}+c\overset{N}{\underset{i=1}{\sum }}\xi_{i}^{\;\;\;2}\\ s.t\;\;y_{i}\left(\omega^{Τ}x_{i}+b\right)\geq 1-\xi_{i},i=1,2,\ldots ,N \end{array}\right.$$

where ω is inertia weight.

Support Vector Machine (SVM) employs a nonlinear mapping function $\Phi :R^{d}\to H$ to transform linearly inseparable samples from the original low-dimensional input space into a higher-dimensional feature space H, where a linear classifier can effectively separate the data. To ensure that inner products in the high-dimensional space correspond to computations in the original space, kernel functions $k\left(x_{i},x_{j}\right)$ are introduced based on general functional theory. Here, $\alpha_{i}$ denotes the Lagrange multipliers. Accordingly, Equation (20) can be reformulated as follows:

(20) $$\left\{\begin{array}{c} Q\left(\alpha \right)=\frac{1}{2}\overset{N}{\underset{i=1}{\sum }}\alpha_{i}\alpha_{j}y_{i}y_{j}k\left(x_{i},x_{j}\right)-\overset{N}{\underset{i=1}{\sum }}\alpha_{i}\\ s.t\;\overset{N}{\underset{i=1}{\sum }}a_{i}y_{i}=0,0\leq a_{i}\leq C,i=1,2,\;\ldots ,N \end{array}\right.$$

In this study, the SVM employs a widely adopted radial basis function (RBF) kernel, defined as follows:

(21) $$k\left(x,y\right)=e^{-\gamma \|x_{i}-x_{j}\|}$$

Here, γ denotes the kernel parameter, which critically influences the classification performance of SVM by determining the effective width of the radial basis function. The overall classification accuracy and computational complexity of an SVM model largely depend on the appropriate selection of two hyperparameters: the penalty coefficient C and the kernel width γ. However, these parameters are typically selected empirically, often leading to suboptimal results and reduced efficiency.

To address this issue, this section introduces a novel hybrid model, OMGOA-SVM, which utilizes the OMGOA to simultaneously optimize C and γ. The enhanced model is subsequently applied to two real-world classification tasks: medical diagnosis and financial forecasting. It comprises two primary stages. In the first stage, OMGOA adaptively tunes the hyperparameters C and γ to improve the performance of the SVM classifier. In the second stage, the optimized SVM is evaluated using 10-fold cross-validation, where nine folds are used for training and the remaining fold for testing, to assess the model’s classification accuracy (ACC).

#### 4.6.1. Performance Metrics

To evaluate the performance of the binary classification model, four standard metrics derived from the confusion matrix were employed:

- True Positive (TP): Instances where the model correctly predicts the positive class.
- False Positive (FP): Instances where the model incorrectly predicts the positive class for a negative sample.
- False Negative (FN): Instances where the model incorrectly predicts the negative class for a positive sample.
- True Negative (TN): Instances where the model correctly predicts the negative class.

Among these metrics, Accuracy (ACC) is defined as the proportion of correctly classified instances (both positive and negative) relative to the total number of predictions. It provides an overall measure of the model’s classification effectiveness.

(22) $$ACC=\frac{TP+TN}{TP+FP+FN+TN}$$

Specificity evaluates the ability of the binary classification model to correctly identify negative (normal) instances, reflecting its effectiveness in distinguishing non-target cases.

(23) $$Specificity=\frac{TN}{FP+TN}$$

Sensitivity measures the model’s ability to correctly identify positive (abnormal) instances, thereby assessing its effectiveness in detecting target conditions or events.

(24) $$Sensitivity=\frac{TP}{TP+FN}$$

The Matthews Correlation Coefficient (MCC) was employed to provide a comprehensive evaluation of the classification model’s performance, offering a more balanced and informative measure than simple accuracy metrics [41].

(25) $$MCC=\frac{TP\times TN-FP\times FN}{\sqrt{\left(TP+FP\right)\times \left(TP+FN\right)\times \left(TN+FP\right)\times \left(TN+FN\right)}}$$

In the subsequent experiments, the proposed OMGOA-SVM model was compared with several other SVM variants, including GOA-SVM, LPO-SVM [42], SBOA-SVM [43], and CPO-SVM [44], using the aforementioned evaluation metrics.

To ensure a fair and consistent comparison, all experiments were conducted under identical settings. Specifically, the swarm size and number of iterations for OMGOA, LPO, SBOA, and CPO algorithms were both fixed at 20 and 50, respectively. The search ranges for the penalty parameter C and kernel width γ were uniformly set to $\left[2^{-5},2^{5}\right]$. Moreover, to mitigate the influence of differing feature scales, all datasets were normalized to the range [0, 1] prior to classification.

#### 4.6.2. Lithology Predictor

Accurate identification of subsurface lithologies or rock types is fundamental for geoscientists engaged in the exploration of underground resources, particularly within the oil and gas sector. Lithology, which denotes the composition and characteristics of subsurface rocks, typically includes categories such as sandstone, claystone, marl, limestone, and dolomite. Various subsurface measurements, notably wireline petrophysical logs, serve as valuable data sources for lithology identification. However, the traditional interpretation of these logs is often labor-intensive, repetitive, and time-consuming.

This study aims to leverage machine learning classification techniques to predict lithology directly from petrophysical log data, providing an efficient and automated approach that addresses these challenges by utilizing logs as effective proxies for lithological properties.

The dataset comprises 118 wells distributed across the South and North Viking Graben, encompassing a geologically diverse region ranging from Permian evaporites in the south to the deeply buried Brent delta facies. Analysis of the provided training data reveals that the offshore Norwegian lithology is predominantly characterized by shales and shaly sediments. These are followed in abundance by sandstones, limestones, marls, and tuffs.

The provided dataset comprises well logs, interpreted lithofacies, and lithostratigraphic information for over 90 wells from offshore Norway. The well logs include identifiers such as well name (WELL), measured depth, and spatial coordinates (x, y, z) corresponding to the wireline measurements. The dataset also contains various petrophysical log measurements, including CALI, RDEP, RHOB, DHRO, SGR, GR, RMED, RMIC, NPHI, PEF, RSHA, DTC, SP, BS, ROP, DTS, DCAL, and MUDWEIGHT. Detailed descriptions of these abbreviations are provided in the figure below.

Table 7 presents a comprehensive comparison of the classification performance of the proposed OMGOA-SVM model against four alternative SVM-based classifiers: GOA-SVM, LPO-SVM, SBOA-SVM, and CPO-SVM. The evaluation metrics considered include Accuracy (ACC), Sensitivity, Specificity, and Matthews Correlation Coefficient (MCC). For each metric, both the average (Avg) and standard deviation (Std) across 10 experimental runs (#1 to #10) are reported, providing insights into both central tendency and variability of model performance. In this experiment, the parameter settings of the support vector machine (SVM) are as follows: the penalty parameter C is set to 10, the kernel function type is selected as radial basis function (RBF), and its kernel parameter γ is set to 0.01. To ensure the stability of the model and avoid overfitting, tenfold cross validation was used for parameter tuning. Specifically, the optimal combination of C and γ is obtained through grid search method, where the value range of C is [0.1, 1, 10, 100], and the value range of γ is [0.001, 0.01, 0.1, 1].

The OMGOA-SVM consistently achieves the highest average accuracy of 0.825, surpassing the other methods whose accuracies range between 0.775 and 0.804. Its standard deviation of 0.081 indicates relatively stable performance across different test folds. Similarly, OMGOA-SVM attains the best average sensitivity (0.756) and specificity (0.845), demonstrating superior ability in correctly identifying positive and negative classes respectively. In terms of MCC, which offers a balanced measure of prediction quality accounting for true and false positives and negatives, OMGOA-SVM again leads with an average value of 0.618, reflecting its comprehensive predictive capability.

Notably, individual fold results reveal that, while performance varies across different subsets, OMGOA-SVM maintains generally higher or comparable metric values compared to other models, indicating robustness. The observed lower standard deviations further confirm its consistent behavior. This comparative analysis underscores the effectiveness of the OMGOA optimization strategy in tuning SVM parameters, thereby enhancing classification accuracy and reliability relative to competing approaches.

## 5. Conclusions and Future Works

In this work, we presented OMGOA, a novel GOA variant that incorporates the Outpost mechanism and a multi-population enhanced mechanism to address the shortcomings of the original GOA. The Outpost mechanism strengthens local search by focusing exploitation efforts near high-quality solutions, while the multi-population strategy enhances global search capabilities and prevents premature convergence by maintaining diverse search dynamics across multiple interacting subpopulations. Through extensive experimental validation, including ablation studies, scalability tests, and comparisons on the CEC2017 benchmark set, OMGOA consistently outperformed both the original GOA and several advanced optimization algorithms. Moreover, its effectiveness was further demonstrated in a real-world lithology prediction task, where OMGOA-based SVM models exhibited superior classification accuracy. Overall, OMGOA offers a promising and robust optimization framework for solving complex, high-dimensional, and real-world problems. Future research may explore the integration of adaptive parameter control and hybrid learning strategies to further enhance its performance. The disadvantage of OMGOA is that its optimization performance has not been validated in multiple practical problems, which hinders its further development.

Future research can explore several promising directions. For example, the proposed OMGOA may be further enhanced by hybridizing it with other emerging metaheuristic algorithms to improve its optimization performance. Additionally, extending OMGOA to tackle multi-objective optimization problems and applying it to tasks such as image segmentation represent worthwhile avenues of investigation. Another prospective line of inquiry involves analyzing how the numerical degradation of chaotic systems in digital computation environments influences the performance of metaheuristic-based optimization methods.

## Figures and Tables

**Table 1:** IEEE CEC 2017 functions.

Function Equation	Dim	Optimum
$f_{1}\left(x\right)=x_{1}^{2}+10^{6}\sum_{i=2}^{D}x_{i}^{2}$	30	100
$f_{2}\left(x\right)=\sum_{i=1}^{D}\left|x_{i}^{2}\right|$	30	200
$f_{3}\left(x\right)=\sum_{i=1}^{D}x_{i}^{2}+{\left(\sum_{i=1}^{D}0.5x_{i}^{2}\right)}^{2}+{\left(\sum_{i=1}^{D}0.5x_{i}^{2}\right)}^{4}$	30	300
$f_{4}\left(x\right)=\sum_{i=1}^{D-1}\left(100{\left(x_{i}^{2}-x_{i+1}\right)}^{2}+{\left(x_{i}-1\right)}^{2}\right)$	30	400
$f_{5}\left(x\right)=\sum_{i=1}^{D}\left(x_{i}^{2}-10\cos\left(2\pi x_{i}\right)+10\right)$	30	500
$f_{6}\left(x\right)=g\left(x_{1},x_{2}\right)+g\left(x_{2},x_{3}\right)+\ldots +g\left(x_{D-1},x_{D}\right)+g\left(x_{D},x_{1}\right)$ $g\left(x,y\right)=0.5+\frac{\left(\sin^{2}\left(\sqrt{x^{2}+y^{2}}\right)-0.5\right)}{{\left(1+0.001\left(x^{2}+y^{2}\right)\right)}^{2}}$	30	600
$f_{7}\left(x\right)=\min\left(\sum_{i=1}^{D}{\left({\overset{⌢}{x}}_{i}-\mu_{0}\right)}^{2},dD+s\sum_{i=1}^{D}{\left({\overset{⌢}{x}}_{i}-\mu_{1}\right)}^{2}\right)+10\left(D-\sum_{i=1}^{D}\cos\left(2\pi {\overset{⌢}{z}}_{i}\right)\right)$	30	700
$f_{8}\left(x\right)=\sum_{i=1}^{D}\left(z_{i}^{2}-10\cos\left(2\pi z_{i}\right)+10\right)+f_{13}$	30	800
$f_{9}\left(x\right)=\sin^{2}\left(\pi w_{1}\right)+\sum_{i=1}^{D}{\left(w_{i}-1\right)}^{2}\left[1+10\sin^{2}\left(\pi w_{i}+1\right)\right]+{\left(w_{D}-1\right)}^{2}\left[1+\sin^{2}\left(2\pi w_{D}\right)\right]$	30	900
$f_{10}\left(x\right)=418.9829\times D-\sum_{i=1}^{D}g\left(z_{i}\right),\;z_{i}=x_{i}+4.209687462275036e+002$	30	1000
$f_{11}\left(x\right)=\sum_{i=1}^{D}{\left(10^{6}\right)}^{\frac{i-1}{D-1}}x_{i}^{2}$	3	1100
$f_{12}\left(x\right)=10^{6}x_{1}^{2}+\sum_{i=2}^{D}x_{i}^{2}$	3	1200
$f_{13}\left(x\right)=-20\exp\left(-0.2\sqrt{\frac{1}{D}\sum_{i=1}^{D}x_{i}^{2}}\right)-\exp\left(\frac{1}{D}\sum_{i=1}^{D}\cos\left(2\pi x_{i}\right)\right)+20+e$	3	1300
$f_{14}\left(x\right)=\sum_{i=1}^{D}\left(\sum_{k=0}^{kmax}\left[a^{k}\cos\left(2\pi b^{k}(x+0.5\right))\right]\right)-D\sum_{k=0}^{kmax}\left[a^{k}\cos\left(2\pi b^{k}.0.5\right)\right]$	4	1400
$f_{15}\left(x\right)=\sum_{i=1}^{D}\frac{x_{i}^{2}}{4000}-\prod_{i=1}^{D}\cos\left(\frac{x_{i}}{\sqrt{i}}\right)+1$	4	1500
$f_{16}\left(x\right)=\frac{10}{D^{2}}\prod_{i=1}^{D}{\left(1+i\sum_{j=1}^{32}\frac{\left|2^{j}x_{i}-round\left(2^{j}x_{i}\right)\right|}{2^{j}}\right)}^{\frac{10}{D^{1.2}}}-\frac{10}{D^{2}}$	4	1600
$f_{17}\left(x\right)={\left|\sum_{i=1}^{D}x_{i}^{2}-D\right|}^{1/4}+\left(0.5\sum_{i=1}^{D}x_{i}^{2}+\sum_{i=1}^{D}x_{i}\right)/D+0.5$	5	1700
$f_{18}\left(x\right)={\left|{\left(\sum_{i=1}^{D}x_{i}^{2}\right)}^{2}-{\left(\sum_{i=1}^{D}x_{i}\right)}^{2}\right|}^{1/4}+\left(0.5\sum_{i=1}^{D}x_{i}^{2}+\sum_{i=1}^{D}x_{i}\right)/D+0.5$	5	1800
$f_{19}\left(x\right)=f_{7}\left(f_{4}\left(x_{1},x_{2}\right)\right)+f_{7}\left(f_{4}\left(x_{2},x_{3}\right)\right)+\cdots +f_{7}\left(f_{4}\left(x_{D-1},x_{D}\right)\right)+f_{7}\left(f_{4}\left(x_{D},x_{1}\right)\right)$	5	1900
$f_{20}\left(x\right)={\left[\frac{1}{D-1}\sum_{i=1}^{D-1}\left(\sqrt{s_{i}}\cdot \left(\sin\left(50.0s_{i}^{0.2}\right)+1\right)\right)\right]}^{2},s_{i}=\sqrt{x_{i}^{2}+x_{i+1}^{2}}$	6	2000
$f_{21}\left(x\right)=f_{1}\left(M\left(x-o_{1}\right)\right)+f_{21}^{\;\;\;*}$	3	2100
$f_{22}\left(x\right)=f_{2}\left(M\left(x-o_{2}\right)\right)+f_{22}^{\;\;\;*}$	3	2200
$f_{23}\left(x\right)=f_{3}\left(M\left(x-o_{3}\right)\right)+f_{23}^{\;\;\;*}$	4	2300
$f_{24}\left(x\right)=f_{4}\left(M\left(\frac{2.048\left(x-o_{4}\right)}{100}\right)+1\right)+f_{24}^{\;\;\;*}$	4	2400
$f_{25}\left(x\right)=f_{5}\left(M\left(x-o_{5}\right)\right)+f_{25}^{\;\;\;*}$	5	2500
$f_{26}\left(x\right)=f_{20}\left(M\left(\frac{2.048\left(x-o_{6}\right)}{100}\right)\right)+f_{26}^{\;\;\;*}$	5	2600
$f_{27}\left(x\right)=f_{7}\left(M\left(\frac{600\left(x-o_{7}\right)}{100}\right)\right)+f_{27}^{\;\;\;*}$	6	2700
$f_{28}\left(x\right)=f_{8}\left(\frac{5.12\left(x-o_{8}\right)}{100}\right)+f_{28}^{\;\;\;*}$	6	2800
$f_{29}\left(x\right)=f_{9}\left(M\left(\frac{5.12\left(x-o_{9}\right)}{100}\right)\right)+f_{29}^{\;\;\;*}$	3	2900
$f_{30}\left(x\right)=f_{30}\left(M\left(\frac{1000\left(x-o_{10}\right)}{100}\right)\right)+f_{30}^{\;\;\;*}$	3	3000

**Table 2:** IEEE CEC 2022 functions.

Functions	Describe	$f_{i}$
f1	Rotated Zakharov	300
f2	Rotated Rosenbrock	400
f3	Expanded Schaffer’s f6	600
f4	Non-Continuous Restrain	800
f5	Levy function	900
f6	Hybrid function	1800
f7	Hybrid function	2000
f8	Hybrid function	2200
f9	Composition function	2300
f10	Composition function	2400
f11	Composition function	2600
f12	Composition function	2700

**Table 3:** Ablation analysis.

Algorithm	Rank	+/=/−	AVG
OMGOA	1	~	1.8
OGOA	2	8/3/19	2.8
MGOA	3	18/5/7	2.5
GOA	4	15/3/12	2.9

**Table 4:** Scalability tests in three dimensions.

	Dim	30		50		100	
	Metric	OMGOA	GOA	OMGOA	GOA	OMGOA	GOA
**Func 1**	Average	9.6544E+03	2.0719E+09	4.9951E+03	9.4197E+09	7.3983E+03	4.6969E+10
	Std	8.2970E+03	1.2911E+09	6.7658E+03	4.6188E+09	6.6813E+03	1.1329E+10
**Func 2**	Average	9.6154E+12	1.4109E+32	6.3023E+23	4.1631E+62	1.1633E+69	6.9198E+126
	Std	3.0840E+13	5.9718E+32	3.4179E+24	2.2802E+63	5.5411E+69	3.7863E+127
**Func 3**	Average	6.7784E+03	3.2272E+04	2.8304E+04	8.1979E+04	1.3629E+05	2.0650E+05
	Std	3.0449E+03	1.0902E+04	6.5742E+03	1.6183E+04	1.7561E+04	2.2376E+04
**Func 4**	Average	4.9188E+02	5.6697E+02	5.0693E+02	1.3467E+03	6.7096E+02	4.5123E+03
	Std	2.9775E+01	5.4096E+01	5.3408E+01	5.3588E+02	6.0517E+01	1.0093E+03
**Func 5**	Average	6.3865E+02	6.0169E+02	8.0247E+02	7.1614E+02	1.2685E+03	1.1204E+03
	Std	3.3990E+01	2.8615E+01	3.9169E+01	3.8068E+01	7.2399E+01	5.6630E+01
**Func 6**	Average	6.1407E+02	6.0786E+02	6.3547E+02	6.1662E+02	6.5558E+02	6.3487E+02
	Std	9.0603E+00	4.8834E+00	1.0859E+01	5.7965E+00	4.7890E+00	5.6141E+00
**Func 7**	Average	8.9554E+02	8.6942E+02	1.1618E+03	1.0451E+03	2.1794E+03	1.8975E+03
	Std	4.5217E+01	4.9829E+01	1.1463E+02	7.4468E+01	2.2714E+02	1.1805E+02
**Func 8**	Average	9.2238E+02	8.7957E+02	1.1032E+03	1.0223E+03	1.5885E+03	1.4628E+03
	Std	1.9283E+01	1.3931E+01	4.8232E+01	3.5415E+01	1.0850E+02	7.3771E+01
**Func 9**	Average	3.1047E+03	1.9553E+03	1.0046E+04	7.0314E+03	2.2550E+04	2.6541E+04
	Std	1.2182E+03	6.4199E+02	2.5073E+03	2.9472E+03	1.1486E+03	1.0968E+04
**Func 10**	Average	4.7856E+03	3.8642E+03	7.2000E+03	6.7377E+03	1.5513E+04	1.4765E+04
	Std	5.8539E+02	4.3079E+02	9.9839E+02	7.7824E+02	7.4991E+02	1.3677E+03
**Func 11**	Average	1.2336E+03	1.8038E+03	1.3668E+03	5.3601E+03	2.5582E+03	4.7428E+04
	Std	5.1988E+01	6.1567E+02	6.9752E+01	2.1302E+03	2.6735E+02	1.1113E+04
**Func 12**	Average	7.7527E+05	7.5541E+07	2.7063E+06	1.3096E+09	8.0200E+06	8.4485E+09
	Std	6.2805E+05	9.6453E+07	2.3197E+06	1.8338E+09	3.8543E+06	4.2430E+09
**Func 13**	Average	1.9697E+05	1.6582E+07	6.4760E+04	1.2606E+08	6.2171E+04	1.0143E+09
	Std	8.0617E+05	3.9994E+07	5.1037E+04	1.0785E+08	3.1854E+04	8.8827E+08
**Func 14**	Average	4.0321E+04	2.3825E+05	8.6009E+04	6.5216E+05	4.1612E+05	5.2014E+06
	Std	4.8977E+03	3.7515E+05	6.2355E+04	6.8071E+05	1.6010E+05	3.8989E+06
**Func 15**	Average	2.7666E+03	3.7331E+05	2.7889E+04	2.7835E+07	3.9627E+04	2.1011E+08
	Std	2.1363E+03	9.3947E+05	1.1444E+04	1.1094E+08	2.9937E+04	3.6221E+08
**Func 16**	Average	1.7772E+05	2.3767E+03	3.4557E+03	2.8582E+03	6.1340E+03	5.8063E+03
	Std	1.1614E+05	2.4430E+02	5.0033E+02	3.8188E+02	8.0273E+02	6.3565E+02
**Func 17**	Average	5.6614E+03	1.9572E+03	3.1105E+03	2.7269E+03	5.9729E+03	4.8923E+03
	Std	3.0003E+03	1.6833E+02	3.4512E+02	2.8575E+02	7.9455E+02	9.3835E+02
**Func 18**	Average	2.4021E+03	1.4865E+06	6.7694E+05	5.6188E+06	8.5258E+05	4.3156E+06
	Std	1.8400E+02	2.5730E+06	3.3266E+05	6.6294E+06	3.3218E+05	2.8652E+06
**Func 19**	Average	2.3957E+03	2.6154E+06	2.6279E+04	7.0771E+06	1.1636E+04	1.3006E+08
	Std	4.4267E+01	7.4123E+06	1.1565E+04	1.9040E+07	1.2975E+04	2.1899E+08
**Func 20**	Average	2.3007E+03	2.3577E+03	3.1415E+03	2.7589E+03	5.1451E+03	4.2834E+03
	Std	1.2373E+00	1.5643E+02	3.1161E+02	2.1393E+02	6.4993E+02	4.2542E+02
**Func 21**	Average	2.7599E+03	2.3882E+03	2.5469E+03	2.5029E+03	3.0414E+03	2.9340E+03
	Std	3.4134E+01	3.3274E+01	5.4655E+01	3.2799E+01	1.2679E+02	7.9164E+01
**Func 22**	Average	2.9507E+03	4.4209E+03	7.1379E+03	8.5861E+03	1.9879E+04	1.8040E+04
	Std	3.9636E+01	1.6321E+03	3.8485E+03	7.3823E+02	1.2933E+03	3.0434E+03
**Func 23**	Average	2.8903E+03	2.7456E+03	3.0194E+03	2.9615E+03	3.6495E+03	3.4885E+03
	Std	1.2045E+01	3.7873E+01	6.8125E+01	4.0778E+01	1.6156E+02	6.7436E+01
**Func 24**	Average	3.6057E+03	2.9369E+03	3.1553E+03	3.1629E+03	4.1844E+03	4.1405E+03
	Std	1.2124E+03	6.1639E+01	5.0881E+01	9.5689E+01	2.0426E+02	1.0695E+02
**Func 25**	Average	3.2773E+03	2.9932E+03	3.0695E+03	3.6087E+03	3.3405E+03	6.3031E+03
	Std	3.7509E+01	5.9165E+01	3.3171E+01	2.6305E+02	4.7277E+01	7.5532E+02
**Func 26**	Average	3.2170E+03	4.5370E+03	3.0389E+03	6.4067E+03	1.7077E+04	1.4457E+04
	Std	1.9431E+01	3.9846E+02	7.6055E+02	4.3337E+02	7.1817E+03	1.1656E+03
**Func 27**	Average	3.8402E+03	3.2484E+03	3.6260E+03	3.5763E+03	3.7511E+03	4.0170E+03
	Std	2.7825E+02	2.0759E+01	8.3670E+01	1.0590E+02	1.2260E+02	1.4984E+02
**Func 28**	Average	5.2491E+04	3.3914E+03	3.3461E+03	4.3159E+03	3.5338E+03	8.0941E+03
	Std	8.8109E+04	7.3941E+01	4.2472E+01	4.2040E+02	3.7578E+01	1.3082E+03
**Func 29**	Average	9.6544E+03	3.7755E+03	4.6991E+03	4.3908E+03	7.0208E+03	7.9534E+03
	Std	8.2970E+03	1.8496E+02	3.7106E+02	3.6453E+02	5.6226E+02	6.3398E+02
**Func 30**	Average	9.6154E+12	7.2129E+06	2.0286E+06	8.3159E+07	1.9587E+06	5.7162E+08
	Std	3.0840E+13	6.2514E+06	9.1926E+05	3.8995E+07	2.9391E+06	6.1185E+08
**+/-/=**	~	~	16/10/4	~	15/13/2	~	16/10/4

**Table 5:** Experiments comparing OMGOA with alternative competing algorithms on the IEEE CEC 2017 benchmark functions.

Algorithm	Rank	+/=/−	AVG
OMGOA	1	~	1.33
HGWO	8	30/0/0	8.23
WEMFO	4	28/0/2	4.50
mSCA	12	30/0/0	11
SCADE	11	30/0/0	10.3
CCMWOA	7	30/0/0	8.17
QCSCA	6	27/0/3	5.40
BWOA	9	30/0/0	8.43
CCMSCSA	2	21/1/8	2.93
CLACO	3	25/0/5	3.87
BLPSO	5	25/3/2	5.33
GCHHO	10	29/0/1	8.50

**Table 6:** Experiments comparing OMGOA with alternative competing algorithms at IEEE CEC 2022 benchmark functions.

Algorithm	Rank	+/=/−	AVG
OMGOA	1	~	2.25
HGWO	10	10/1/1	7.83
WEMFO	5	9/2/1	6.00
mSCA	6	10/0/2	6.50
SCADE	11	10/2/0	9.33
CCMWOA	9	9/3/0	7.67
QCSCA	3	6/0/6	4.75
BWOA	7	9/2/1	6.58
CCMSCSA	12	10/1/1	9.67
CLACO	4	4/2/6	5.33
BLPSO	8	8/2/2	6.83
GCHHO	2	7/2/3	4.25

**Table 7:** Comparison of classification performance of OMGOA-SVM with other classifiers.

Indicator	Value	OMGOA-SVM	GOA-SVM	LPO-SVM	SBOA-SVM	CPO-SVM
	Avg	0.825	0.804	0.792	0.779	0.775
	Std	0.081	0.076	0.081	0.090	0.113
	#1	0.750	0.875	0.833	0.875	0.875
	#2	0.792	0.792	0.792	0.792	0.792
	#3	0.958	0.833	0.958	0.958	0.958
ACC	#4	0.875	0.875	0.792	0.750	0.750
	#5	0.833	0.792	0.750	0.833	0.708
	#6	0.792	0.792	0.708	0.708	0.708
	#7	0.750	0.750	0.792	0.792	0.750
	#8	0.917	0.833	0.708	0.708	0.708
	#9	0.875	0.875	0.875	0.667	0.917
	#10	0.708	0.625	0.708	0.708	0.583
	Avg	0.756	0.729	0.705	0.714	0.739
	Std	0.187	0.143	0.192	0.158	0.134
	#1	0.917	0.917	0.917	0.917	0.917
	#2	0.786	0.714	0.786	0.786	0.786
	#3	0.889	0.778	0.889	0.889	0.889
Sensitivity	#4	0.778	0.778	0.556	0.778	0.667
	#5	0.667	0.556	0.667	0.667	0.556
	#6	0.833	0.833	0.833	0.833	0.722
	#7	0.286	0.429	0.286	0.571	0.571
	#8	0.900	0.700	0.700	0.700	0.700
	#9	0.833	0.833	0.833	0.417	0.917
	#10	0.667	0.750	0.583	0.583	0.667
	Avg	0.845	0.836	0.836	0.798	0.785
	Std	0.138	0.143	0.143	0.186	0.137
	#1	0.917	0.917	0.917	0.917	0.917
	#2	0.786	0.714	0.714	0.786	0.786
	#3	0.889	0.778	0.778	0.889	0.889
Specificity	#4	0.778	0.778	0.778	0.778	0.667
	#5	0.667	0.556	0.556	0.667	0.556
	#6	0.833	0.833	0.833	0.833	0.722
	#7	0.286	0.429	0.429	0.571	0.571
	#8	0.900	0.700	0.700	0.700	0.700
	#9	0.833	0.833	0.833	0.417	0.917
	#10	0.667	0.750	0.750	0.583	0.667
	Avg	0.618	0.577	0.542	0.526	0.523
	Std	0.190	0.171	0.204	0.207	0.523
	#1	0.530	0.753	0.676	0.753	0.753
	#2	0.580	0.608	0.580	0.580	0.580
	#3	0.913	0.644	0.913	0.913	0.913
MCC	#4	0.730	0.730	0.547	0.497	0.467
	#5	0.639	0.547	0.467	0.639	0.365
	#6	0.476	0.476	0.178	0.178	0.348
	#7	0.312	0.348	0.470	0.476	0.395
	#8	0.829	0.657	0.410	0.410	0.410
	#9	0.753	0.753	0.753	0.385	0.833
	#10	0.418	0.258	0.430	0.430	0.169

## Data Availability

The numerical and experimental data used to support the findings of this study are included within the article.
